# Efficacy and Safety of Standardized Ethanol Extract of Purple Perilla (*Perilla frutescens* Britton var. *acuta* Kudo) Leaves in Cognitive Impairment: A Randomized, Double-Blind, Placebo-Controlled Clinical Trial

**DOI:** 10.3390/nu18060960

**Published:** 2026-03-18

**Authors:** Hyang-Im Baek, Jong Cheon Joo, Sung-Kyu Kim, Mi-Houn Park, Gun Hee Cho, Lei Shen, Soo Jung Park

**Affiliations:** 1 Department of Food Science & Nutrition, Woosuk University, Wanju 55338, Republic of Korea; hyangim100@gmail.com; 2 Department of Sasang Constitutional Medicine, College of Korean Medicine, Wonkwang University, Iksan 54538, Republic of Korea; jcjoo2000@hanmail.net; 3 Borambio Co., Ltd., Cheonan 31116, Republic of Korea; skkim@borambio.com (S.-K.K.); pmh@borambio.com (M.-H.P.); joo2413@borambio.com (G.H.C.); 4 Aerospace Center Hospital, Beijing 100049, China; 5 Department of Sasang Constitutional Medicine, College of Korean Medicine, Woosuk University, Jeonju 55338, Republic of Korea

**Keywords:** *Perilla frutescens* Britton var. *acuta* Kudo, purple perilla, cognitive function, cognitive impairment, functional food, clinical trial

## Abstract

**Objectives**: This randomized, double-blind, placebo-controlled 12-week clinical trial evaluated the efficacy and safety of a standardized ethanol extract of purple perilla leaves (*Perilla frutescens* Britton var. *acuta* Kudo; PE) in adults with cognitive impairment. **Methods**: Subjects who met the inclusion criteria were randomly assigned in a 1:1 ratio to one of two groups and received PE (*n* = 50, 500 mg/day) or placebo (*n* = 50) for 12 weeks. The primary efficacy outcomes included cognitive function, which was assessed by the Korean mini-mental status examination–2 (K–MMSE–2) and the Alzheimer’s disease assessment scale–cognitive subscale (ADAS–Cog), and plasma amyloid β (Aβ) and brain-derived neurotrophic factor (BDNF) levels, which were measured as secondary biochemical markers. The safety biomarkers were also assessed before and after the intervention. **Results**: After 12 weeks of intervention, the K–MMSE–2 total score, the K–MMSE–2 subdomain scores (attention and calculation and language), the ADAS–Cog total score, and the ADAS–Cog subscale scores (word recall, commands, delayed word recall, naming, word recognition, and recall instructions) showed statistically significant between-group improvements compared with the placebo group. Improvements were observed in both groups, whereas the magnitude of cognitive enhancement was greater in the PE group, indicating an effect beyond placebo-related responses. No statistically significant between-group differences were observed in plasma Aβ or BDNF levels. The safety evaluation found no clinically significant changes. **Conclusions**: Twelve-week administration of PE significantly improved cognitive outcomes without safety concerns, suggesting its potential as a standardized botanical ingredient for supporting cognitive function in individuals with early cognitive impairment.

## 1. Introduction

Alzheimer’s disease (AD) is a neurodegenerative disease that is characterized by the progressive and irreversible deterioration of cognitive and functional abilities, leading to difficulties in daily life [1]. AD is a major cause of dementia [2,3], and with the aggravated aging problem, the prevalence of AD is gradually increasing. According to Alzheimer’s Disease International estimates in 2018, about 50 million people worldwide suffer from dementia, and the most recent data indicate that by 2050, the global prevalence of dementia will triple [4,5]. The United States reported that the proportion of deaths related to AD is rising, increasing by 89% between 2000 and 2014 [6]. The direct and indirect costs of AD-related medical care also place a heavy burden on society. At present, the treatment of AD mainly includes cholinesterase inhibitors and the N-methyl-D-aspartate receptor antagonist memantine, which enhances cognitive treatment, and the serotonin 2A (5–HT2A) receptor inverse agonist pimavanserin, which improves neuropsychiatric symptoms, and a multidomain lifestyle-based intervention [3]. Recently, most researchers have been devoted to disease-modifying therapies, among which amyloid β and tau biology are the two most common targets. Although much research has been done on many aspects of AD, there are few effective treatments and interventions that change the course of the disease, and some therapeutic drugs are commonly accompanied by side effects, such as nausea, dizziness, and anorexia [7]. Therefore, it is essential to find effective alternative treatments.

Traditional herbal medicines have long been applied to maintain or restore cognitive health, largely owing to their multi-component and multi-target actions that can simultaneously modulate several neurobiological pathways [8,9,10]. *Perilla frutescens* (L.) belongs to the family Lamiaceae and is widely cultivated as an edible crop in China, Japan, and South Korea. It has been reported that different plant parts of *P. frutescens* contain numerous bioactive secondary metabolites, such as terpenoids, flavonoids, alkaloids, steroids, quinones, and phenolic compounds, exhibiting high antioxidant, anticancer, anti-inflammatory, and antimicrobial activities [11]. Among its botanical varieties, the purple-leaf variety is particularly rich in anthocyanins, rosmarinic acid, and other phenolic compounds that contribute to potent antioxidant, anti-inflammatory, and neuroprotective effects [12]. This variety has been traditionally used in East Asian medicine to manage respiratory conditions, including coughs, asthma, and allergic disorders [13,14]. In addition to its traditional applications, its bioactive constituents have recently attracted attention for their potential relevance to cognitive function.

Previous studies have shown that the main active components of purple perilla exhibit neuroprotective effects and can be used as a dietary supplement to prevent neurodegenerative diseases, such as AD and cerebral ischemia disorders [15]. Cho et al. [16] showed that in an amyloid mouse model of AD (5XFAD), standardized ethanol extract of purple perilla leaves (*Perilla frutescens* Britton var. *acuta* Kudo; PE) blocked amyloid β (Aβ) aggregate-induced memory impairment, and the oral administration of PE for approximately 1 month improved memory impairment in 5XFAD mice, suggesting that PE might be a promising therapeutic candidate for AD. In addition, many studies have shown that the extract of purple perilla and RA improve cognitive function and memory through Aβ aggregation and decomposition regulation and antioxidant and anti-inflammatory effects [17,18,19,20,21,22,23,24]. More recently, PE was reported to alleviate Aβ-induced neuroinflammation by downregulating JNK/NF–κB signaling and enhancing CREB (cAMP response element-binding protein)/BDNF (brain-derived neurotrophic factor)-mediated neurogenesis following daily administration for 7 days in an Aβ-induced AD-like mouse model [25]. Furthermore, the same extract improved learning and memory by enhancing hippocampal synaptic plasticity through the muscarinic acetylcholine receptor (mAChR) and the calcium-permeable AMPA receptor (CP–AMPAR) pathways in scopolamine-induced cognitive dysfunction after the daily oral administration prior to the scopolamine challenge during the behavioral testing period [26]. These preclinical findings provide a mechanistic foundation for the present clinical investigation assessing the cognitive benefits of PE in humans.

To further explore whether these mechanistic pathways are reflected in clinically relevant biological markers, we considered biomarkers associated with synaptic plasticity and amyloid pathology. The BDNF is a key neurotrophin involved in synaptic plasticity, neuronal survival, and learning and memory processes [27], and reduced circulating BDNF levels have been associated with cognitive decline and neurodegenerative disorders [28]. In addition, Aβ is a central pathological hallmark of Alzheimer’s disease [29], and altered plasma Aβ levels have been explored as indicators of amyloid-related pathology and disease progression [30]. Given the anti-amyloidogenic and neuroprotective properties of PE that have been demonstrated in preclinical models, the circulating BDNF and Aβ were selected as mechanistically relevant exploratory biomarkers in the present clinical trial.

Based on previous studies, we learned that the early identification of an at-risk population together with subsequent intervention in the pre-clinical stage might be helpful in slowing or halting the progression of AD [31]. Although rosmarinic acid and PE have demonstrated strong neuroprotective, anti-inflammatory, and anti-amyloidogenic activities in preclinical models, the clinical relevance of these biological effects remains insufficiently validated in well-controlled human trials using chemically standardized extracts. Therefore, we conducted a 12-week, randomized, double-blind, placebo-controlled clinical trial to evaluate whether standardized PE administration improves cognitive function and whether it is well tolerated in older adults with cognitive impairment.

## 2. Materials and Methods

### 2.1. Ethics

The trial was performed in Wonkwang University Jeonju Korean Medicine Hospital from May 2022 to October 2022. The ethical approval for this study was obtained from the Institutional Review Board (IRB) of Wonkwang University Jeonju Korean Medicine Hospital (IRB approval No.: WUJKMH–IRB–2021–0012; approval date: 15 November 2021), which also reviewed and approved the informed consent form prior to participant enrollment. This study was performed according to the Declaration of Helsinki and Good Clinical Practice (GCP) guidelines. The study was conducted in accordance with the principles of the CONSORT guidelines for randomized clinical trials. The trial was retrospectively registered with the Clinical Research Information Service (CRIS) of the Republic of Korea (CRIS No.: KCT0008844; registered on 6 October 2023).

### 2.2. Study Design

This 12-week clinical trial was conducted using a double-blind, randomized controlled design to evaluate the efficacy and safety of PE on cognitive decline. In this study, participants were required to visit a total of four times. The screening was carried out on the first visit (day 28), and the baseline data were collected on the second visit (day 0), followed by a visit every six weeks, that is, visit 3 (day 42 ± 7: follow-up) and visit 4 (day 84 ± 7: end of the study). Informed consent was obtained from the subjects during the screening, and their demographic data and medical history were subsequently recorded. The subjects who met the criteria were rechecked at the baseline, and the AD assessment scale-cognitive subscale (ADAS–Cog; see Section 2.5), brain-derived neurotrophic factor (BDNF), and Aβ were measured at the baseline and after 12 weeks of treatment. During the screening period and at the end of the study, drinking and smoking history, height, weight, and body mass index (BMI) were recorded, and related laboratory tests were performed, including common blood cell counts (e.g., leukocyte, red blood cell, hemoglobin, hematocrit, and platelets), liver function, renal function, lipid profiles, and creatine kinase. The subjects underwent the Korean mini-mental status examination–2 (K–MMSE–2, see Section 2.5), an electrocardiogram, and a pregnancy test at the baseline and after 12 weeks of treatment. The subjects’ medication history, blood pressure, and pulse rate were recorded during each visit. Their dietary intake was monitored, and the Global Physical Activity Questionnaire (GPAQ) was performed at the baseline and the end of the study. During the study period, the subjects maintained their dietary habits, lifestyle, and physical activity as usual.

We used a computer-generated randomization sheet to randomly allocate the enrolled subjects to either the treatment or control group in a 1:1 ratio. All participants, investigators, and study staff remained blind to the group allocation until the completion of the data analysis. Unblinding was allowed if serious adverse events occurred or an emergency arose for the subjects.

### 2.3. Study Participants

The inclusion criteria were as follows: (1) they were aged 55 years or older, (2) K–MMSE–2 of 25–28, (3) they were able to understand and complete the cognitive assessment, and (4) they agreed to sign the written informed consent form.

The subjects were excluded for any of the following: (1) the subjects were currently receiving treatment due to the structured clinical interview of DSM-IV (SCID) axis I disorder during the screening period, or had a history of treatment within 3 years; (2) their BMI was <18.5 or ≥35 kg/m^2^ at screening; (3) they had a history of alcohol abuse or addiction within the previous 3 months; (4) the subjects had acute or chronic cardio-cerebrovascular, a severe mental illness, or liver, kidney and blood system diseases, etc.; (5) the subjects had an allergy to any of the constituents in the study medication; (6) they were administering any other medications, supplements or herbs that had the potential to affect cognition or enhance memory within one month prior to screening; (7) the subjects who had donated whole blood within one month prior to screening or component blood within 2 weeks prior to screening; (8) they had participated in other clinical trials within the three months prior to screening; (9) they had levels of serum aspartate aminotransferase and alanine aminotransferase that were three times more than the upper limit of normal values as seen in kidney function tests showing blood urea nitrogen (BUN) > 2.0 mg/dL; (10) they were pregnant or lactating women; and (11) they were women of childbearing age who did not use the proper methods of contraception.

No sex-based restriction was applied during the recruitment. The higher proportion of female participants likely reflects the voluntary nature of study enrollment and the known epidemiological pattern of cognitive dysfunction [32].

### 2.4. Study Products and Interventions

**Figure 1:**
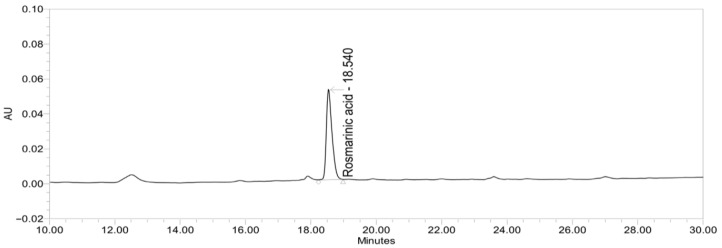
The representative HPLC chromatogram of the standardized PE. The chromatographic profile was obtained using high-performance liquid chromatography (HPLC). Rosmarinic acid, the marker compound used for standardization, was detected at a retention time of approximately 18.5 min. The PE used in the present clinical trial was standardized to contain 12.7 mg/g (80–120%) of rosmarinic acid.

The PE raw material was provided by Boram Bio Co., Ltd. (Cheonan, Republic of Korea), and the material used in this study was manufactured at the production facility of S&D Co., Ltd. (Cheonan, Republic of Korea) in Lot No. 210915-001. The preparation method of the raw material has previously been described [16,25,26]. Dried and pulverized purple perilla leaves (*Perilla frutescens* Britton var. *acuta* Kudo) were extracted using 60% ethanol and subsequently spray-dried. Rosmarinic acid was selected as the marker compound for standardization based on its well-documented neuroprotective, antioxidant, and anti-inflammatory activities, as well as its abundance in purple perilla leaves. The PE raw material was standardized to contain 12.7 mg/g (80–120%) of rosmarinic acid. The chemical profile of the standardized PE was confirmed by high-performance liquid chromatography (HPLC) analysis, and the representative chromatogram identifying rosmarinic acid is shown in Figure 1.

Using this standardized PE raw material (Lot No. 210915–001), the active study product (Lot No. SFJB211001) and the placebo product (Lot No. SFJP211001) were manufactured in capsule form at Suheung Co., Ltd. (Cheongju, Republic of Korea). All eligible participants received their assigned interventions: participants were instructed to take two capsules of either the study product (providing 500 mg/day of PE) or the placebo product (containing 0 mg/day of PE) once daily after a meal for 12 weeks. The study product contained the PE as the active ingredient, whereas the placebo product was formulated with inert ingredients lacking known physiological activity, primarily a lactose mixture and crystalline cellulose. Both the PE and placebo capsules were identical in appearance, weight, and other physical properties. The study products were dispensed to participants at each study visit in pre-labeled containers according to the randomization schedule.

### 2.5. Efficacy Outcome Measures

The primary outcomes were the ADAS–Cog, and K–MMSE–2. The secondary outcomes were BDNF and Aβ. These outcomes were assessed before and after the 12-week treatment.

The ADAS–Cog consists of memory, language and praxis assessments [33] and is a commonly used cognitive assessment instrument in clinical trials of AD. The examination time of ADAS–Cog takes 30–45 min, with a higher score indicating a higher degree of deficit. The MMSE is commonly used to assess dementia and estimate the degree of cognitive impairment. Based on this, this study conducted the K–MMSE–2 to evaluate the severity of cognitive function. The total score of K–MMSE–2 was 30 points, including memory registration (3 points), time orientation (5 points), space orientation (5 points), memory recall (3 points), attention and calculation (5 points), language (8 points), and drawing (1 point) [34].

The circulating BDNF and Aβ were included as exploratory secondary biomarkers and were measured to assess potential biological changes associated with PE supplementation.

Fasting venous blood samples were collected after an overnight fast of at least 8 h at the baseline and at the end of the 12-week intervention. Plasma was separated by centrifugation and stored at −70 °C until analysis. The circulating levels of BDNF and Aβ were quantified using commercially available enzyme-linked immunosorbent assay (ELISA) kits according to the manufacturers’ instructions. The BDNF assay was obtained from R&D Systems (Minneapolis, MN, USA), and the Aβ assay was purchased from Immuno-Biological Laboratories (Fujioka, Japan).

### 2.6. Safety Outcome Measures

The safety assessment was performed in this study, including the reporting of adverse events (AEs), vital signs, laboratory tests, ECG, and physical examinations. The vital signs included standardized assessments of systolic blood pressure (SBP), diastolic blood pressure (DBP), pulse rate, and body temperature obtained under standardized resting conditions. The laboratory evaluations were performed on fasting blood and urine samples obtained at the baseline and at the end of the intervention. The analyses comprised hematology, serum biochemistry, and urinalysis. In addition, treatment compliance (counting the remaining capsules) was assessed during the third and fourth visits.

### 2.7. Statistical Analysis

The sample size was calculated based on the change in K–MMSE–2 before and after intervention in the PE and placebo groups. The calculation referenced a previously published study of comparable design [35]. Considering an anticipated dropout rate of 20%, a total of 100 participants were required, with 50 individuals assigned to each group in a 1:1 randomization ratio.

The analyses were performed using SPSS version 27.0 (IBM Corp., Armonk, NY, USA). Continuous data are expressed as the mean ± standard deviation (SD), whereas categorical variables are summarized as frequencies and percentages. The primary efficacy analysis was conducted in the per-protocol set (PPS). Additionally, a full analysis set (FAS), defined according to the intention-to-treat principle, was analyzed to assess the robustness of the findings. The safety assessments were analyzed using the safety set, which was defined as all of the individuals who had administered at least one dose of the study product.

Within-group differences were analyzed using either the paired *t*-test or the Wilcoxon signed-rank test, according to data normality. The between-group comparisons of the changes from the baseline were performed using the independent *t*-test or the Mann–Whitney U-test, depending on the data distribution. The categorical data were evaluated using the Chi-square or Fisher’s exact test, as appropriate. The significance was statistically significant at the level *p* < 0.05.

The primary endpoints were prespecified as the total scores of K-MMSE-2 and ADAS–Cog. Subscale analyses were performed as exploratory assessments and should be interpreted accordingly.

## 3. Results

### 3.1. Baseline Characteristics of Participants

**Figure 2:**
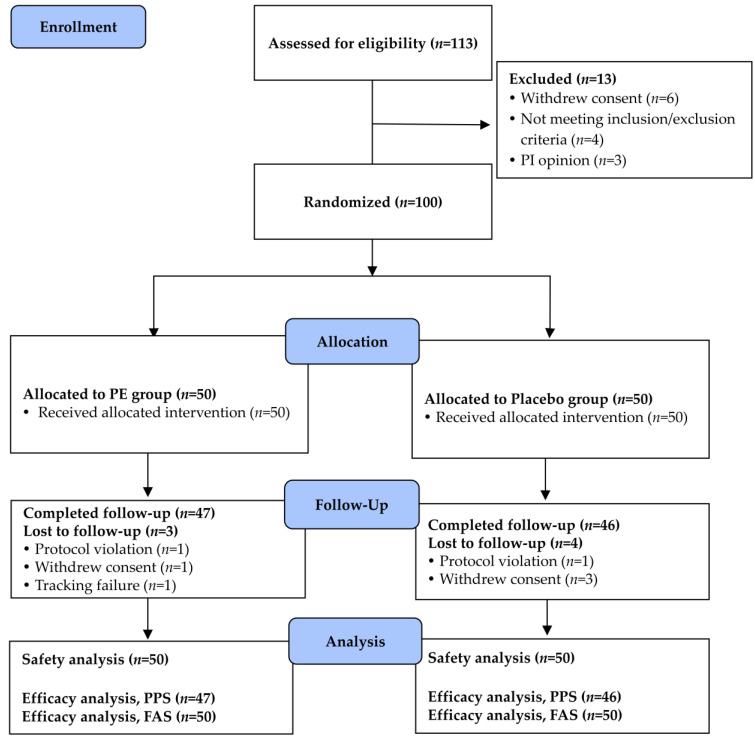
A CONSORT flow diagram of participant disposition. The diagram shows the number of participants enrolled, allocated, followed, and analyzed, in accordance with the CONSORT 2010 guidelines.

A total of 113 subjects were screened, 100 of whom met the inclusion/exclusion criteria and were selected and assigned to the PE and the placebo group. During the 12-week intervention period, a total of seven participants discontinued the study. In the PE group, three individuals were withdrawn, one due to a protocol deviation related to the eligibility criteria, one owing to withdrawal of consent, and one due to failing to follow up. In the placebo group, four participants discontinued participation, one because of a prohibited concomitant medication and three due to a withdrawal of consent. As a result, 93 participants (PE: *n* = 47; placebo: *n* = 46) completed the study according to the protocol criteria (Figure 2).

Table 1 summarizes the demographic characteristics of all participants. There were no significant differences between groups in the baseline demographic characteristics, including age, sex distribution, height, weight, BMI, temperature, SBP, DBP, pulse rate, alcohol consumption, and smoking. In addition, there was no significant difference in the baseline K–MMSE–2 and ADAS–Cog total score between the groups, so randomization was successful.

### 3.2. Efficacy Outcomes

**Figure 3:**
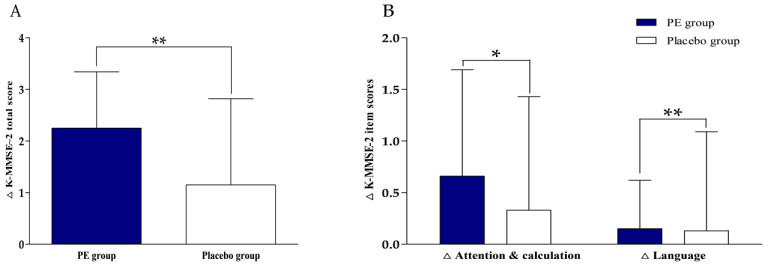
The change in K–MMSE–2 scores after 12 weeks of intervention. (**A**) The change in K–MMSE–2 total score from the baseline at 12 weeks. (**B**) The change in K–MMSE–2 subdomain scores (attention and calculation, and language) from the baseline at 12 weeks. The values are presented as mean ± SD (PE: *n* = 47; placebo: *n* = 46). The between-group comparisons of the changes from the baseline were analyzed using the Mann–Whitney U-test. Δ indicates the change from the baseline. * *p* < 0.05, ** *p* < 0.01 vs. placebo group.

The biomarkers for efficacy evaluation were measured before intervention and 12 weeks after intervention. The K–MMSE–2 results are shown in Table 2 and Figure 3. After 12 weeks of intervention, K–MMSE–2 total score increased by +2.25 ± 1.09 points in the PE group and +1.15 ± 1.67 points in the placebo group. Within-group analyses demonstrated significant improvements from the baseline in both groups, with a significantly greater increase observed in the PE group (between-group, *p* = 0.001). In exploratory subdomain analyses, significant between-group differences were observed in attention and calculation (*p* = 0.027) and language (*p* = 0.001).

**Figure 4:**
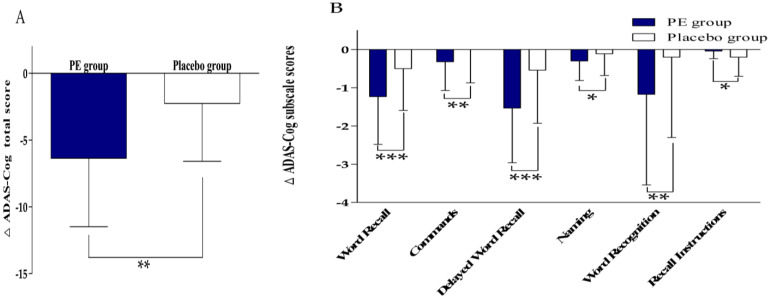
The change in ADAS–Cog scores after 12 weeks of intervention. (**A**) The change in ADAS–Cog total score from the baseline at 12 weeks. (**B**) The change in ADAS–Cog subscale scores (word recall, commands, delayed word recall, naming, word recognition, and recall instructions) from the baseline at 12 weeks. The values are presented as mean ± SD (PE: *n* = 47; placebo: *n* = 46). The between-group comparisons of the changes from the baseline were analyzed using the Mann–Whitney U-test. Δ indicates the change from the baseline. * *p* < 0.05, ** *p* < 0.01, *** *p* < 0.001 vs. placebo group.

The ADAS–Cog results are summarized in Table 3 and Figure 4. After 12 weeks of intervention, the ADAS–Cog total score decreased by −6.36 ± 5.12 points in the PE group and −2.26 ± 4.33 points in the placebo group. Within-group analyses showed significant reductions from the baseline in both groups, with a significantly greater improvement observed in the PE group (between-group *p* = 0.001). In exploratory subscale analyses, significant between-group differences were observed in word recall, commands, delayed word recall, naming, word recognition, and recall instructions (*p* < 0.001, 0.005, <0.001, 0.016, 0.008, 0.045, respectively). The comprehension subscale also showed a trend toward a between-group difference (*p* = 0.080).

Additional analyses using the FAS yielded results that were consistent with those of the PPS analysis, with statistically significant between-group differences for the primary cognitive outcomes maintained (Supplementary Table S1).

The amyloid β and BDNF levels, as determined by blood tests, are shown in Table 4. The comparison of the Amyloid β and BDNF levels before and after 12 weeks of intervention showed no statistically significant difference between the two intervention groups.

### 3.3. Safety Outcomes

A safety evaluation was conducted to evaluate the safety of the test product. During the intervention, AEs were reported in five participants (two in PE, three in placebo). There was no statistically significant difference in the incidence of AEs between the two groups (*p* > 0.05). In addition, no AE had a causal relationship with the study product. No serious AEs were observed in either group.

The laboratory profile results are shown in Table 5. Total bilirubin decreased in the placebo group, so there was a significant difference between the two groups. However, it was within the normal range, and there were no clinically significant changes. In addition, there were no significant differences when comparing safety evaluation indicators between the groups, including vital signs and electrocardiograms.

Taken together, these results suggest that the investigational product exhibited good safety levels and was well-tolerated throughout the study period.

## 4. Discussion

This randomized, double-blind, placebo-controlled clinical trial evaluated the efficacy and safety of PE in adults with cognitive impairment. Daily administration of 500 mg PE for 12 weeks significantly improved both the total and subdomain scores of K–MMSE–2 and ADAS–Cog compared to the placebo, suggesting its potential efficacy in enhancing cognitive function, and confirming its safety in humans.

The clinical indicator for the transition between normal aging and early dementia is cognitive impairment. Compared to normal subjects, subjects with cognitive impairment generally have a higher rate of progression to dementia over a relatively short period of time [36]. Therefore, cognitive impairment is proposed as the best intervention step to improve cognitive function [37]. Thus, in our study, we conducted a clinical trial targeting cognitive impairment and found that cognitive function was improved, which may have implications for early intervention in individuals at risk of dementia.

MMSE is the most widely used standardized test tool in dementia screening [38]. It takes about 10 min to evaluate various cognitive domains, so it can screen for cognitive impairment within a short period of time and is practical for continuous and routine use. The MMSE was developed by Folstein et al. [39], and has been translated and validated in many countries and languages [39,40]. Recently, the Korean mini-mental state examination, 2nd Edition (K–MMSE–2) was released, and verified by Kang et al. [41]. The K–MMSE–2 total score indicates overall cognitive function, while each item represents a domain-specific function [42]. In previous studies, a change of three points or more in MMSE was set as an important change in improving cognitive function [43]. In this study, among subjects whose K–MMSE–2 changed by more than three points, 46.81% of the test group and 23.91% of the placebo group improved, showing a significant improvement in cognitive function in the PE group. In this study, PE supplementation resulted in a 2.25-point increase in K–MMSE–2 over 12 weeks. Although the intervention period was relatively short, the observed improvement suggests potential cognitive benefits, which warrant further long-term investigation.

ADAS–Cog is considered the gold standard to evaluate the efficacy of various anti-dementia treatments [44,45], and is known to be particularly sensitive to treatment response in patients with cognitive impairment or early dementia [46]. ADAS–Cog is an assessment tool originally developed to accurately detect dementia patients with cognitive dysfunction and to measure the degree of functional decline in dementia patients over time [47,48]. The ADAS–Cog 13-item scale [33] is a form in which the number cancellation task and delayed free recall task are added to ADAS–Cog 11. The ADAS–Cog–13 score ranges from 0 to 85, with a higher score indicating higher severity [49]. Previous studies [49,50] have shown that the responsiveness of the treatment for cognitive impairment was better than the ADAS–Cog–13, which in turn, was better than the ADAS–Cog–11. Because this study evaluated the efficacy of PE for cognitive impairment, ADAS–Cog 13 was used. Previous studies have confirmed the reliability and validity of ADAS–Cog, and it is known to have a negative correlation with MMSE [51]. In this study, due to PE intervention, ADAS–Cog was statistically significantly decreased while K–MMSE–2 was significantly increased, showing consistent results with previous studies.

Although several human studies have investigated the cognitive effects of *P. frutescens* seed oil or mixed formulae containing species-level materials [52,53,54,55], no prior human study has specifically examined the cognitive effects of standardized PE. Purple perilla is a widely consumed aromatic herb with high nutritional and phytochemical value. Unlike seed-derived oil or species-level preparations, the PE used in this study was derived exclusively from the purple-leaf variety, which contains abundant anthocyanins, rosmarinic acid, and other phenolic compounds associated with antioxidant and neuroprotective activity [56,57]. The purple-leaf type, commonly referred to as Ja–so–yeop in Korea, has been traditionally used in East Asian medicine for centuries to relieve respiratory and gastrointestinal symptoms, including common colds, coughs, nausea, and bronchial discomfort [58]. Beyond these traditional applications, accumulating preclinical evidence demonstrates that purple perilla leaves exhibit diverse pharmacological properties, such as antiallergic, anti-amyloidogenic, antibacterial, anti-inflammatory, hypolipidemic, and antitumor effects, which are largely attributed to their rich anthocyanin and phenolic profiles [17,59,60,61,62]. These multifunctional biological actions highlight the therapeutic potential of the purple perilla chemotype and underscore the significance of the present trial, which provides the first clinical evidence that demonstrates that a standardized extract prepared solely from purple perilla leaves can improve cognitive function in humans.

In the present trial, 12-week supplementation with PE significantly improved cognitive performance, as reflected by reductions in ADAS–Cog scores and increases in K–MMSE–2 scores. These clinical findings may be compatible with neurobiological mechanisms previously reported in preclinical models. However, the direct modulation of specific pathways cannot be confirmed based on the present clinical data [16,17,18,19,20,21,22,24,25,26,63]. The leaf extract of purple perilla showed an anti-amyloidogenic effect on Aβ aggregation and degradation in PC12 cells, protecting the cells [17,18], and reduced NO production in LPS-stimulated BV2 microglial cells [18]. In 5XFAD transgenic mice, PE attenuated hippocampal Aβ deposition and neuroinflammation, leading to improvements in memory-related behavioral performance [16].

Rosmarinic acid (RA), a major phenolic constituent abundant in purple perilla leaves, has also been widely reported to display antioxidant and anti-inflammatory activities in various *P. frutescens*-derived extracts [20,21,64]. Given that oxidative stress and neuroinflammation are closely associated with cognitive decline, the presence of RA in the standardized extract may contribute to the clinical improvements observed in this study.

Recent mechanistic investigations further suggest that PE downregulates JNK and NF–κB signaling pathways while activating the CREB–BDNF axis in experimental models [25]. Although these findings derive primarily from animal studies, they offer a biologically plausible explanation for the cognitive benefits observed in our clinical population, particularly in domains relevant to early cognitive impairment. In addition, modulation of cholinergic and glutamatergic signaling pathways has been reported in scopolamine-induced models [26], supporting the potential role of PE in enhancing synaptic plasticity. Importantly, the lack of significant changes in the circulating Aβ and BDNF levels suggests that the observed cognitive improvements may not be directly reflected in these peripheral biomarkers within the relatively short intervention period. Within the biomarker cascade framework of Alzheimer’s disease, functional cognitive changes may precede detectable alterations in peripheral biomarkers, particularly in early-stage populations and over short intervention periods [65,66]. Taken together, these findings suggest that PE may confer cognitive benefits that are biologically plausible based on preclinical evidence. However, the absence of significant changes in the circulating Aβ and BDNF levels indicates that definitive conclusions regarding specific molecular pathways cannot be drawn from this study.

In the present study, although PE administration significantly improved cognitive performance across multiple neuropsychological assessments, the circulating Aβ and BDNF levels did not show statistically significant between-group differences. Importantly, this finding does not diminish the clinical relevance of the observed cognitive benefits. Rather, it may reflect both biological and methodological considerations. In the present study, although PE administration significantly improved cognitive performance across multiple neuropsychological assessments, the circulating Aβ and BDNF levels did not show statistically significant between-group changes. This apparent dissociation is not unexpected and may reflect both biological and methodological factors. First, peripheral Aβ measures reflect complex production and clearance dynamics and may only weakly track short-term changes in central amyloid pathology, particularly in early-stage cognitive impairment and in the absence of highly sensitive ratio-based assays or extended follow-up periods [66,67,68]. Consistent with this notion, a previous 12-month clinical trial using *P. frutescens* seed oil reported cognitive improvement accompanied by changes in antioxidant capacity rather than early shifts in amyloid-related biomarkers [52,53,54,55]. Second, peripheral BDNF levels are known to exhibit substantial inter-individual variability and are influenced by multiple non-disease-related factors, including physical activity, sleep, platelet-related release, and metabolic status, which may obscure modest treatment-related effects over a 12-week intervention period [69,70]. Taken together, these findings suggest that cognitive improvements induced by PE may precede detectable changes in circulating biomarkers. In accordance with this, future studies employing longer intervention durations, larger sample sizes, and broader mechanistic panels—such as inflammatory cytokines, oxidative stress indices, and synaptic or neuronal injury markers—are warranted to more comprehensively elucidate the biological pathways underlying PE-associated cognitive benefits [71].

In our study, cognitive function improved in both the PE group and the placebo group. Placebos have traditionally been regarded as inert substances used to control for nonspecific effects in clinical trials and treatments [72]. However, growing evidence indicates that placebo responses are driven by complex psychological and neurobiological mechanisms involving expectation, conditioning, and treatment context, all of which can influence clinical outcomes [73]. Thus, although a placebo represents a dummy intervention, it can nevertheless elicit measurable improvements through expectancy-related effects [72]. In addition, repeated cognitive assessments may introduce learning or practice effects, particularly in short-term trials [74,75]. Familiarity with test procedures may contribute to score improvements independent of true cognitive change. In this study, while both groups exhibited improvements, the magnitude of cognitive enhancement was significantly greater in the PE group, suggesting that the observed effects were not solely attributable to placebo responses. Therefore, the PE appears to exert a genuine cognitive-enhancing effect beyond expectancy-driven improvements. Placebo-related cognitive improvements are frequently observed in short-term supplementation-based trials targeting mild cognitive impairment, typically ranging from approximately 0.5–1.5 points for MMSE-based measures and 1–2 points for ADAS–Cog over 12–24 weeks [35,73,76,77,78,79]. In the present study, the placebo group demonstrated a +1.15-point improvement in K-MMSE-2 and a −2.26-point reduction in the ADAS–Cog score, both of which fall within the range reported in previous trials. However, it is important to note that the magnitude of improvement observed in the PE group (+2.25 points in K-MMSE-2 and −6.36 points in ADAS–Cog) exceeded the typical placebo-associated changes, resulting in statistically significant between-group differences. These findings further support that the cognitive benefits observed with PE supplementation extend beyond the nonspecific placebo responses.

Purple perilla is a purple-leaf chemotype that has traditionally been used for centuries in East Asian countries as both a culinary herb and a medicinal plant. Its long history of dietary and medicinal use supports its safety as a natural food resource [80]. In the present study, 12-week administration with PE was well tolerated, with no clinically meaningful abnormalities observed in adverse events, clinical laboratory parameters, vital signs, or electrocardiographic assessments. These findings indicate that within the tested dosage and duration, PE is safe for human administration.

This study demonstrated that a 12-week PE intervention improved overall cognitive function; however, several limitations should be acknowledged. First, although the sample size was calculated based on previous comparable trials, the number of participants who completed the study was relatively small, which limits the generalizability of the findings. Larger, adequately powered studies are therefore warranted to confirm and extend these results. Second, the 12-week intervention period was relatively short compared to other studies investigating *P. frutescens* preparations. For example, a 12-month clinical trial using *P. frutescens* seed oil reported significant improvement in cognitive performance and antioxidant capacity [52,53,54,55]. Moreover, prior longitudinal studies have shown that changes in ADAS–Cog scores occur gradually, with follow-up durations of up to 3 years required to detect meaningful differences [81,82,83,84,85]. Because our study only evaluated outcomes after 12 weeks, the long-term effects of PE administration could not be determined. Thus, future investigations with extended intervention and follow-up periods are needed to better assess the durability of cognitive benefits. Third, since the brain is highly susceptible to oxidative damage, cognitive decline has been associated with reduced plasma antioxidant capacity and elevated oxidative stress [86,87]. Although preclinical studies have demonstrated the antioxidant activity of PE [19,20,21,24], antioxidant biomarkers were not assessed in this clinical trial; therefore, its in vivo antioxidant efficacy could not be confirmed. Further studies should include oxidative stress-related parameters to clarify whether the cognitive improvement observed is mediated by the antioxidant properties of PE. Fourth, subscale analyses were exploratory in nature, and no adjustment for multiple comparisons was applied. In accordance with this, these results should be interpreted in the context of exploratory analyses.

Nevertheless, this study has the strength of being the first clinical trial to demonstrate the cognitive-enhancing efficacy of standardized PE in humans, supported by a well-controlled, randomized, double-blind, placebo-controlled design. These findings provide meaningful clinical evidence supporting the potential anti-dementia effects of PE.

## 5. Conclusions

This 12-week randomized, double-blind, placebo-controlled clinical trial evaluated the efficacy and safety of standardized PE for supporting cognitive function in individuals with cognitive impairment. PE supplementation (500 mg/day) resulted in statistically significant improvements in the K–MMSE–2 total score and subdomain scores (attention and calculation and language), as well as the ADAS–Cog total score and multiple subscales (word recall, commands, delayed word recall, naming, word recognition, and recall instructions), compared with the placebo group. No clinically relevant safety concerns were observed during the intervention period. Collectively, these findings suggest that standardized PE may serve as a promising botanical ingredient for the nutritional support of cognitive function in early cognitive impairment.

## Figures and Tables

**Table 1:** The baseline demographic characteristics of participants.

		PE Group (*n* = 50)	Placebo Group (*n* = 50)	*p*-Value ^1^
Age (years)		64.96 ± 6.03	65.60 ± 5.86	0.681
Sex (male/female, %)		5 (10.0)/45 (90.0)	7 (14.0)/43 (86.0)	0.760 ^2^
Height (cm)		157.06 ± 5.99	156.56 ± 6.75	0.557
Weight (kg)		62.21 ± 8.48	59.25 ± 7.66	0.098
BMI (kg/m^2^)		25.14 ± 3.01	24.16 ± 2.65	0.143
SBP (mmHg)		125.28 ± 10.05	121.52 ± 10.59	0.052
DBP (mmHg)		75.10 ± 7.25	72.36 ± 8.87	0.671
Pulse rate (beats/minute)		70.06 ± 8.20	71.84 ± 10.67	0.970
Temperature (°C)		36.43 ± 0.23	36.43 ± 0.24	0.435
Smoking	Yes	0 (0.0)	1 (2.0)	1.000 ^2^
	No	50 (100.0)	49 (98.0)	
	cigarettes/day	-	5.00 ± 0.00	
Alcohol	Yes	7 (14.0)	12 (24.0)	0.308 ^2^
	No	43 (86.0)	38 (76.0)	
	units/week	4.50 ± 3.52	4.02 ± 3.44	
K–MMSE–2 total score		26.62 ± 0.67	26.54 ± 0.65	0.525
ADAS–Cog total score		15.98 ± 5.00	17.56 ± 4.89	0.063

The values are presented as mean ± SD or number (%) ^1^ and were analyzed using the Mann–Whitney U-test between the groups ^2^ and using Fisher’s exact test between the groups.

**Table 2:** The changes in K–MMSE–2 before and after 12 weeks of intervention.

	PE Group (*n* = 47)				Placebo Group (*n* = 46)				*p*-Value ^2^
	Baseline	12 Weeks	Change Value	*p*-Value ^1^	Baseline	12 Weeks	Change Value	*p*-Value ^1^	
K–MMSE–2 total score	26.62 ± 0.68	28.87 ± 1.17	2.25 ± 1.09	<0.001	26.57 ± 0.66	27.72 ± 1.54	1.15 ± 1.67	<0.001	0.001 **
Memory registration	2.98 ± 0.15	3.00 ± 0.00	0.02 ± 0.15	0.317	3.00 ± 0.00	3.00 ± 0.00	-	-	0.323
Time orientation	4.85 ± 0.42	4.96 ± 0.20	0.11 ± 0.48	0.132	4.91 ± 0.28	4.93 ± 0.25	0.02 ± 0.39	0.705	0.806
Space orientation	4.79 ± 0.46	4.83 ± 0.38	0.04 ± 0.59	0.617	4.89 ± 0.38	4.87 ± 0.34	−0.02 ± 0.45	0.739	0.169
Memory recall	1.49 ± 0.69	2.72 ± 0.45	1.23 ± 0.81	<0.001	1.59 ± 0.80	2.26 ± 0.85	0.67 ± 0.97	<0.001	0.142
Attention and calculation	3.72 ± 0.77	4.38 ± 0.77	0.66 ± 1.03	<0.001	3.57 ± 0.83	3.89 ± 1.04	0.33 ± 1.10	0.048	0.027 *
Language	7.85 ± 0.47	8.00 ± 0.00	0.15 ± 0.47	0.038	7.63 ± 0.71	7.76 ± 0.60	0.13 ± 0.96	0.335	0.001 **
Drawing	0.94 ± 0.25	0.98 ± 0.15	0.04 ± 0.29	0.317	0.98 ± 0.15	1.00 ± 0.00	0.02 ± 0.15	0.317	0.183

The values are presented as mean ± SD. ^1^ The values were analyzed using the Wilcoxon signed-rank test between baseline and 12 weeks within each group. ^2^ The values were analyzed using the Mann–Whitney U-test between the groups at the change value. * *p* < 0.05, ** *p* < 0.01 vs. the placebo group.

**Table 3:** The changes in ADAS–Cog before and after 12 weeks of intervention.

	PE Group (*n* = 47)				Placebo Group (*n* = 46)				*p*-Value ^2^
	Baseline	12 Weeks	Change Value	*p*-Value ^1^	Baseline	12 Weeks	Change Value	*p*-Value ^1^	
ADAS–Cog Total Score	16.09 ± 5.06	9.72 ± 4.48	−6.36 ± 5.12	<0.001	17.33 ± 4.81	15.07 ± 5.08	−2.26 ± 4.33	0.002	0.001 **
Word Recall	3.62 ± 1.17	2.38 ± 1.07	−1.23 ± 1.25	<0.001	3.89 ± 0.99	3.39 ± 1.00	−0.50 ± 1.09	0.004	<0.001 ***
Commands	0.55 ± 0.69	0.23 ± 0.43	−0.32 ± 0.75	0.007	0.63 ± 0.61	0.63 ± 0.64	0.00 ± 0.87	1.000	0.005 **
Construction	0.62 ± 0.61	0.47 ± 0.50	−0.15 ± 0.72	0.162	0.70 ± 0.59	0.63 ± 0.53	−0.07 ± 0.65	0.491	0.135
Delayed Word Recall	3.30 ± 1.44	1.77 ± 1.32	−1.53 ± 1.43	<0.001	3.61 ± 1.41	3.07 ± 1.42	−0.54 ± 1.39	0.012	<0.001 ***
Naming	0.38 ± 0.53	0.09 ± 0.28	−0.30 ± 0.51	<0.001	0.48 ± 0.59	0.37 ± 0.57	−0.11 ± 0.57	0.197	0.016 *
Ideational Praxis	0.70 ± 0.55	0.62 ± 0.57	−0.09 ± 0.54	0.285	0.85 ± 0.51	0.67 ± 0.56	−0.17 ± 0.49	0.021	0.200
Orientation	0.26 ± 1.19	0.09 ± 0.28	−0.17 ± 1.24	0.564	0.07 ± 0.33	0.07 ± 0.25	0.00 ± 0.42	1.000	0.290
Word Recognition	3.34 ± 2.05	2.17 ± 1.74	−1.17 ± 2.37	0.002	3.52 ± 2.18	3.33 ± 1.84	−0.20 ± 2.10	0.474	0.008 **
Recall Instructions	0.04 ± 0.20	0.00 ± 0.00	−0.04 ± 0.20	0.157	0.24 ± 0.64	0.04 ± 0.29	−0.20 ± 0.50	0.014	0.045 *
Number Cancelation	1.30 ± 0.93	0.96 ± 0.91	−0.34 ± 1.01	0.022	1.35 ± 0.90	1.33 ± 0.92	−0.02 ± 0.83	0.858	0.167
Spoken Language	0.26 ± 0.44	0.06 ± 0.25	−0.19 ± 0.40	0.003	0.30 ± 0.47	0.17 ± 0.38	−0.13 ± 0.50	0.083	0.175
Word-Finding Difficulty	0.68 ± 0.47	0.21 ± 0.41	−0.47 ± 0.50	<0.001	0.65 ± 0.48	0.37 ± 0.49	−0.28 ± 0.54	0.002	0.383
Comprehension	1.04 ± 0.66	0.68 ± 0.56	−0.36 ± 0.70	0.002	1.04 ± 0.70	1.00 ± 0.60	−0.04 ± 0.76	0.697	0.080

The values are presented as mean ± SD. ^1^ The values were analyzed using the Wilcoxon signed-rank test between the baseline and 12 weeks within each group. ^2^ The values were analyzed using the Mann–Whitney U-test between the groups at the change value. * *p* < 0.05, ** *p* < 0.01, *** *p* < 0.001 vs. placebo group.

**Table 4:** The changes in amyloid β and BDNF levels before and after 12 weeks of intervention.

	PE Group (*n* = 47)				Placebo Group (*n* = 46)				*p*-Value ^2^
	Baseline	12 Weeks	Change Value	*p*-Value ^1^	Baseline	12 Weeks	Change Value	*p*-Value ^1^	
Amyloid β (pg/mL)	12.17 ± 45.84	10.44 ± 33.67	−1.73 ± 13.46	0.840	3.81 ± 2.84	4.30 ± 4.36	0.49 ± 2.36	0.959	0.569
BDNF (pg/mL)	25,391.00 ± 7627.43	25,908.36 ± 7217.72	17.36 ± 5467.07	0.975	25,038.46 ± 6291.31	24,464.70 ± 6020.40	−573.76 ± 6838.40	0.722	0.830

The values are presented as mean ± SD. ^1^ The values were analyzed using the Wilcoxon signed-rank test between the baseline and 12 weeks within each group. ^2^ The values were analyzed using the Mann–Whitney U-test between the groups at the change value.

**Table 5:** The changes in the laboratory profile before and after 12 weeks of intervention.

	PE Group (*n* = 50)				Placebo Group (*n* = 50)				*p*-Value ^2^
	Baseline	12 Weeks	Change Value	*p*-Value ^1^	Baseline	12 Weeks	Change Value	*p*-Value ^1^	
Hematology									
WBC (10^3^/μL)	6.00 ± 1.51	5.88 ± 1.26	−0.12 ± 0.83	0.424	5.94 ± 1.67	5.78 ± 1.14	−0.16 ± 1.48	0.804	0.512
RBC (100^3^/μ)	4.36 ± 0.35	4.36 ± 0.33	0.00 ± 0.15	0.836	13.30 ± 0.91	13.30 ± 0.93	−0.01 ± 0.19	0.553	0.535
Hemoglobin (g/dL)	13.46 ± 1.04	13.48 ± 1.06	0.02 ± 0.46	0.828	4.27 ± 0.34	4.27 ± 0.34	0.01 ± 0.53	0.933	0.865
Hematocrit (%)	40.88 ± 2.72	40.59 ± 2.82	−0.29 ± 1.22	0.066	40.12 ± 2.66	39.88 ± 2.62	−0.24 ± 1.64	0.279	0.992
Platelet (10^3^/μL)	241.52 ± 49.04	238.22 ± 45.73	−3.30 ± 26.47	0.559	233.38 ± 50.80	237.80 ± 50.44	4.42 ± 18.85	0.071	0.130
Biochemistry									
ALP (U/L)	191.76 ± 58.23	197.74 ± 78.20	5.98 ± 67.65	0.627	192.56 ± 50.32	198.96 ± 70.76	6.40 ± 51.98	0.857	0.537
AST (U/L)	24.84 ± 6.39	47.30 ± 152.38	22.46 ± 152.25	0.221	21.42 ± 6.93	22.96 ± 9.48	0.92 ± 5.66	0.202	0.693
ALT (U/L)	21.94 ± 8.06	35.50 ± 89.53	13.56 ± 89.67	0.171	25.54 ± 4.87	26.46 ± 6.29	1.54 ± 8.61	0.321	0.95
Total bilirubin (mg/dL)	0.67 ± 0.19	0.73 ± 0.22	0.05 ± 0.20	0.075	0.77 ± 0.29	0.71 ± 0.26	−0.06 ± 0.16	0.025	0.007
Total protein (g/dL)	6.74 ± 0.41	6.90 ± 0.31	0.16 ± 0.35	0.003	6.79 ± 0.41	6.90 ± 0.33	0.11 ± 0.31	0.018	0.447
Albumin (g/dL)	4.14 ± 0.22	4.20 ± 0.23	0.06 ± 0.23	0.031	4.24 ± 0.23	4.26 ± 0.21	0.02 ± 0.20	0.478	0.197
Gamma-GT (U/L)	22.22 ± 14.87	24.84 ± 23.50	2.62 ± 22.17	0.483	21.20 ± 11.31	24.56 ± 35.32	3.36 ± 27.81	0.734	0.645
BUN (mg/dL)	15.15 ± 4.09	14.78 ± 3.34	−0.37 ± 3.95	0.783	14.98 ± 3.11	14.83 ± 3.48	−0.15 ± 3.34	0.747	0.978
Creatinine (mg/dL)	0.79 ± 0.11	0.81 ± 0.12	0.02 ± 0.09	0.092	0.82 ± 0.15	0.85 ± 0.14	0.02 ± 0.09	0.076	0.991
Total Cholesterol (mg/dL)	195.26 ± 41.55	197.08 ± 43.31	1.82 ± 21.06	0.307	200.48 ± 40.60	196.60 ± 41.23	−3.88 ± 27.60	0.477	0.244
HDL Cholesterol (mg/dL)	54.30 ± 13.99	53.66 ± 13.47	−0.64 ± 9.58	0.826	55.14 ± 12.93	53.56 ± 12.95	−1.58 ± 8.37	0.094	0.134
Triglyceride (mg/dL)	104.54 ± 63.90	108.58 ± 51.87	4.04 ± 47.68	0.333	109.72 ± 53.78	115.74 ± 56.56	6.02 ± 46.23	0.431	0.847
Glucose (mg/dL)	97.06 ± 11.54	98.02 ± 11.32	0.96 ± 8.47	0.256	98.40 ± 10.22	99.72 ± 11.61	1.32 ± 7.44	0.469	0.507
LD (U/L)	202.00 ± 29.24	199.78 ± 40.47	−2.22 ± 36.38	0.087	205.66 ± 30.26	200.30 ± 30.51	−5.36 ± 16.71	0.015	0.374
LDL Cholesterol (mg/dL)	125.18 ± 36.78	125.24 ± 39.39	0.06 ± 17.08	0.835	127.24 ± 38.51	124.28 ± 40.79	−2.96 ± 27.05	0.631	0.807
Creatinine kinase (U/L)	106.66 ± 49.59	100.34 ± 39.22	−6.32 ± 34.11	0.210	116.78 ± 72.61	112.22 ± 61.32	−4.56 ± 78.81	0.537	0.929
Urinalysis									
Specific gravity	1.00 ± 0.14	1.02 ± 0.01	0.02 ± 0.14	0.093	1.02 ± 0.01	1.02 ± 0.01	0.00 ± 0.01	0.289	0.408
pH	5.84 ± 0.91	5.96 ± 0.90	0.12 ± 1.01	0.316	5.71 ± 0.85	5.97 ± 1.02	0.26 ± 0.97	0.020	0.628

The values are presented as mean ± SD. ^1^ The values were analyzed using the Wilcoxon signed-rank test within the groups. ^2^ The values were analyzed using the Mann–Whitney U-test between groups at the change value.

## Data Availability

The datasets that were generated and analyzed during the current study are available from the corresponding author on reasonable request. The data are not publicly available due to privacy and ethical restrictions.

## Supplementary Materials

The following supporting information can be downloaded at https://www.mdpi.com/article/10.3390/nu18060960/s1. Table S1: Changes in efficacy outcomes before and after 12-week intervention (FAS).
