# Supplementary material for: Efficacy and Safety of Standardized Ethanol Extract of Purple Perilla (Perilla frutescens Britton var. acuta Kudo) Leaves in Cognitive Impairment: A Randomized, Double-Blind, Placebo-Controlled Clinical Trial
Source: Nutrients. 2026 Mar 18;18(6):960. doi: 10.3390/nu18060960 (PMC13029764; doi:10.3390/nu18060960)
Supplement: Supplementary file 1 [file nutrients-18-00960-s001.zip › nutrients-4160340-supplementary.pdf]

Table S1: Changes in efficacy outcomes before and after 12-week intervention (FAS).

	PE group (*n*=50)				Placebo group (*n*=50)				*p*-Value [2]
	**Baseline**	**12 weeks**	**Change value**	***p*-Value [1]**	**Baseline**	**12 weeks**	**Change value**	***p*-Value [1]**	
**K–MMSE–2**									
Total score	26.62 ± 0.67	28.74 ± 1.26	2.12 ± 1.19	<0.001	26.54 ± 0.65	27.60 ± 1.54	1.06 ± 1.63	<0.001	<0.001***
Memory registration	2.98±0.14	3.00±0.00	0.02±0.14	0.317	3.00±0.00	3.00±0.00	–	–	0.317
Time orientation	4.84±0.42	4.94±0.24	0.10±0.46	0.132	4.92±0.27	4.94±0.24	0.02±0.38	0.705	0.438
Space orientation	4.80±0.45	4.84±0.37	0.04±0.57	0.617	4.88±0.39	4.86±0.35	-0.02±0.43	0.739	0.332
Memory recall	1.50±0.68	2.66±0.52	1.16±0.84	<0.001	1.54±0.79	2.16±0.89	0.62±0.95	<0.001	0.052
Attention & calculation	3.70±0.76	4.32±0.79	0.62±1.01	<0.001	3.60±0.83	3.90±1.02	0.30±1.05	0.048	0.075
Language	7.86±0.45	8.00±0.00	0.14±0.45	0.038	7.62±0.70	7.74±0.60	0.12±0.92	0.335	<0.001***
Drawing	0.94±0.24	0.98±0.14	0.04±0.28	0.317	0.98±0.14	1.00±0.00	0.02±0.14	0.317	0.175
**ADAS–Cog**									
Total score	15.98 ± 5.00	10.00 ± 4.57	-5.98 ± 5.18	<0.001	17.56 ± 4.89	15.48 ± 5.26	-2.08 ± 4.20	0.002	<0.001***
Word Recall	3.60±1.16	2.44±1.09	-1.16±1.25	<0.001	3.84±1.00	3.38±0.99	-0.46±1.05	0.004	<0.001***
Commands	0.56±0.67	0.26±0.44	-0.30±0.74	0.007	0.70±0.65	0.70±0.68	0.00±0.83	1.000	<0.001***
Construction	0.62±0.60	0.48±0.50	-0.14±0.70	0.162	0.72±0.57	0.66±0.52	-0.06±0.62	0.491	0.066
Delayed Word Recall	3.26±1.41	1.82±1.30	-1.44±1.43	<0.001	3.62±1.38	3.12±1.41	-0.50±1.34	0.012	<0.001***
Naming	0.36±0.53	0.08±0.27	-0.28±0.50	<0.001	0.52±0.58	0.42±0.57	-0.10±0.54	0.197	<0.001***
Ideational Praxis	0.70±0.54	0.62±0.57	-0.08±0.53	0.285	0.82±0.52	0.66±0.56	-0.16±0.47	0.021	0.295
Orientation	0.24±1.15	0.08±0.27	-0.16±1.20	0.564	0.08±0.34	0.08±0.27	0.00±0.40	1.000	0.605
Word Recognition	3.30±2.01	2.20±1.71	-1.10±2.31	0.002	3.60±2.15	3.42±1.84	-0.18±2.02	0.474	0.002**
Recall Instructions	0.04±0.20	0.00±0.00	-0.04±0.20	0.157	0.24±0.62	0.06±0.31	-0.18±0.48	0.014	0.016*
Number Cancellation	1.30±0.93	0.98±0.91	-0.32±0.98	0.022	1.40±0.95	1.38±0.97	-0.02±0.80	0.858	0.100
Spoken Language	0.26±0.44	0.08±0.27	-0.18±0.39	0.003	0.28±0.45	0.16±0.37	-0.12±0.48	0.083	0.373
Word-Finding Difficulty	0.70±0.46	0.26±0.44	-0.44±0.50	<0.001	0.66±0.48	0.40±0.49	-0.26±0.53	0.002	0.481
Comprehension	1.04±0.64	0.70±0.54	-0.34±0.69	0.002	1.08±0.70	1.04±0.60	-0.04±0.73	0.697	0.031*
Amyloid β (pg/mL)	11.85±44.44	10.22±32.65	-1.63±13.05	0.840	3.73±2.74	4.19±4.20	0.46±2.26	0.959	0.574
BDNF (pg/mL)	25608.18±7571.83	26094.50±7170.96	486.32±5298.52	0.975	24808.92±6303.47	24281.06±6037.23	-527.86±6555.23	0.722	0.855

Values are presented as mean ± SD. [1] Analyzed using Wilcoxon singed-rank test between baseline and 12 weeks within each group. [2] Analyzed using Mann Whitney U-test between the groups at change value. **p* <0.05, ***p* <0.01, ****p* <0.001 vs. placebo group.
